# An Integrated Multi-Omics Study of Mammalian Skeletal, Cardiac, and Smooth Muscles

**DOI:** 10.3390/ijms27010242

**Published:** 2025-12-25

**Authors:** Shengguo Tang, Kaiming Wang, Dongfang Li, Yanna Ma, Liangyuan Peng, Shuchao Liao, Haiming Ma, Hongjiang Wei

**Affiliations:** 1 Faculty of Animal Science and Technology, Yunnan Agricultural University, Kunming 650500, China; 15802587908@163.com (S.T.); dfli0927@163.com (D.L.); 2 Institute of Yunnan Circular Agricultural Industry, Pu’er 665000, China; 15874851690@163.com (Y.M.); 15023692717@163.com (L.P.); 17666225547@163.com (S.L.); 3 Yunnan Province Key Laboratory for Porcine Gene Editing and Xenotransplantation, Yunnan Agricultural University, Kunming 650500, China; 4 College of Animal Science and Technology, Hunan Agricultural University, Changsha 410128, China; 15116529648@stu.hunau.edu.cn (K.W.); mahaiming@hunau.edu.cn (H.M.)

**Keywords:** smooth muscle, cardiac muscle, skeletal muscle, gene, metabolome

## Abstract

Muscle tissue, as a major tissue type, is classified by its structure and function into smooth, cardiac, and skeletal muscle. However, comprehensive studies on the evolutionary conservation of molecular differences among these three muscle tissues have been limited. In this study, we employed pigs and mice as models to perform multi-omics profiling (transcriptome, proteome, and metabolome) of these three muscle tissues in order to define their molecular landscapes. Furthermore, we characterized skeletal muscle metabolic heterogeneity. We identified 207 genes enriched in striated muscle, including poorly characterized genes such as *LRRC2* and *PPP1R14C*. Distinct sets of genes and metabolites, conserved between the two species, were specifically enriched in each tissue: skeletal muscle (121 genes and 6 metabolites), cardiac muscle (57 genes and no specific metabolites), and smooth muscle (349 genes and 11 metabolites). Notably, the currently unannotated gene *LRRC20* was most enriched in skeletal muscle, followed by cardiac muscle, and showed negligible expression in smooth muscle, suggesting its potential as a functional research target. Within skeletal muscle, 14 fast-twitch and 6 slow-twitch fiber-enriched metabolites were identified. In particular, 10-Deacetylbaccatin III was enriched in skeletal muscle and, more specifically, highly enriched in fast-twitch fibers, marking it as a promising and novel research target. These results provide a resource for research in both medicine and agricultural science.

## 1. Introduction

The contraction and relaxation of muscle tissue underlie all motor functions in mammals [1,2,3]. Muscle tissue is composed of muscle cells, which are mainly long and fibrous and therefore called muscle fibers. Muscle tissue is classified by its structure and function into smooth, cardiac, and skeletal muscle [4]: smooth muscle is found in internal organs, such as the intestine [5]; cardiac muscle is found exclusively in the heart [6]; and skeletal muscle is typically connected to the bones of the limbs [7]. Unlike smooth muscle, both cardiac and skeletal muscle are striated and share more similarities [8]. Thus, systematically understanding the genetic and metabolic differences among the three types of muscle tissue is crucial for various areas of research, including regeneration, development, and xenotransplantation, as these muscle tissues constitute the basic units of movement and metabolism in the body. However, comparative studies in this area remain limited.

The proper function of these muscle tissues relies not only on their contractile properties but also on their capacity for maintenance and repair. The regenerative potential of muscle tissues varies markedly in mammals. Smooth muscle retains significant regenerative ability; skeletal muscle exhibits limited regeneration that is mediated by satellite cells; and adult cardiomyocytes rarely proliferate, resulting in largely absent regeneration. The systematic identification of regeneration-associated genes across all three muscle tissues has therefore become a research priority. *TECRL* (trans-2,3-enoyl-CoA reductase-like), an endoplasmic reticulum protein, serves as a representative example. It is most enriched in cardiac muscle, followed by skeletal muscle, and found in negligible amounts in smooth muscle. Functionally, *TECRL* plays opposing roles in the two striated muscle types. While its loss impairs cardiac function in mice, demonstrating its necessity [9], the specific ablation of *TECRL* in satellite cells, in stark contrast, enhances skeletal muscle repair and promotes growth [10]. Another gene, *FBXO40*, encodes a muscle-specific F-box protein and is upregulated during denervation atrophy. It is a striated muscle-specific gene and is not expressed in smooth muscle. *FBXO40* loss in mice increases IGF-1 levels, leading to increased body and skeletal muscle mass [11], a conserved effect also observed in pigs [12]. The existence of other functionally important genes such as *TECRL* and *FBXO40*, however, has not been systematically explored. Moreover, the metabolic differences among muscle tissues are also poorly understood. To this end, we propose a systematic, multi-species, multi-omics analysis to identify differentially expressed genes and metabolites across all three muscle tissues. The findings of this study will advance our understanding of muscle biology and provide insights for regenerative medicine and agriculture.

Muscle fiber types are defined by the expression of distinct myosin heavy chain (MYH) isoforms. For instance, smooth muscle, which is not under voluntary control, expresses the *MYH11* gene, encoding its characteristic myosin heavy chain. Both cardiac and skeletal muscle are striated muscles but differ fundamentally in their fiber-type composition. Cardiac muscle expresses characteristic markers of slow-twitch fibers (e.g., *MYH7*); in comparison, skeletal muscle comprises a mixture of fast-twitch (e.g., *MYH1*, *MYH2* and *MYH4*) and slow-twitch (e.g., *MYH7*) fibers. Accounting for approximately 40% of mammalian body weight [7], skeletal muscle also functions as a secretory organ, releasing myokines (such as *MSTN*, a protein enriched in skeletal muscle) that can remotely regulate other organs [13]. While epigenetic regulation of muscle fiber type is well-studied [14], skeletal muscle evolutionarily conserved metabolomic features remain poorly explored.

In order to fill this knowledge gap, we profiled the multi-omics landscapes (transcriptome, proteome, and metabolome) of cardiac, skeletal, and smooth muscle in pig and mouse models. With a particular focus on skeletal muscle, we further performed metabolomics to directly contrast fast-twitch-dominant and slow-twitch-dominant fibers to uncover the metabolic differences that underpin their physiological diversity. Our study serves as a reference for the systemic understanding of muscle tissues.

## 2. Results

### 2.1. Integration of Transcriptomic and Proteomic Analysis of Cardiac, Smooth, and Skeletal Muscles in Pigs and Mice

**Figure 1:**
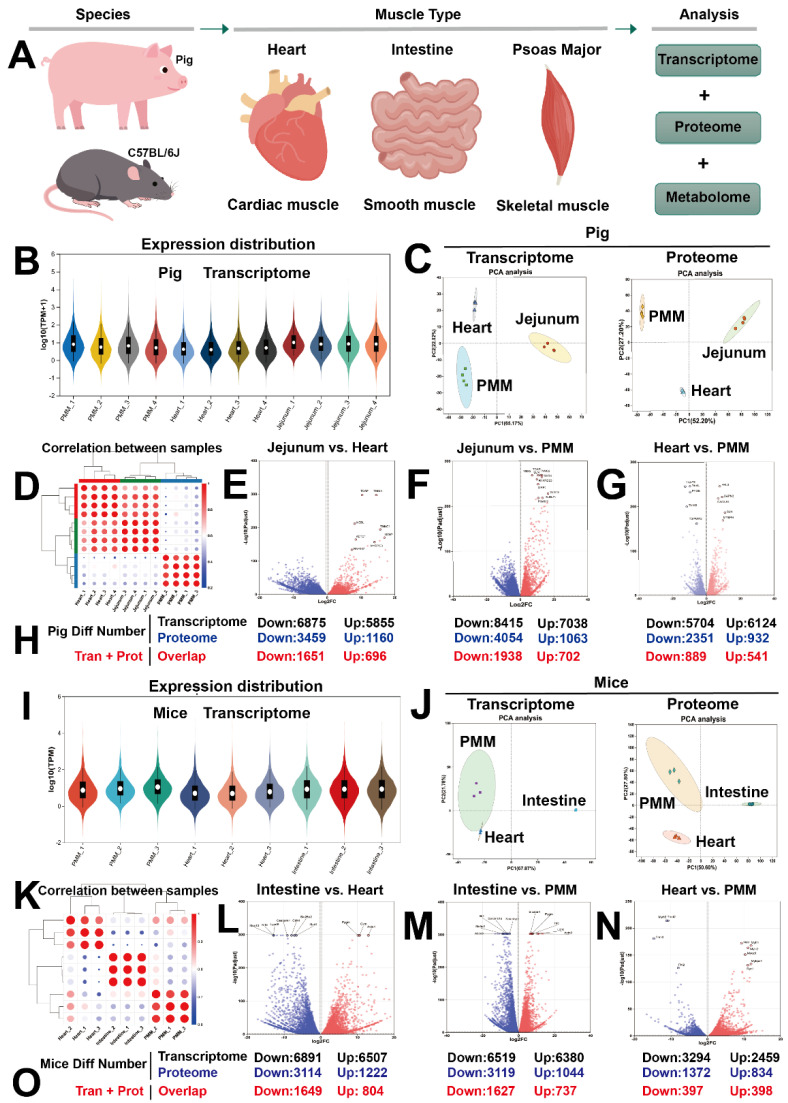
Multi-omics profiling of three muscle tissues in pigs and mice. (**A**) Experimental design. Tissue samples (intestinal, cardiac, and skeletal muscle) were collected from adult male pigs (180 days old) and mice (8 weeks old) for transcriptomic (|FC| ≥ 1.2, *p* < 0.05), proteomic (|FC| ≥ 2, *p* < 0.05), and metabolomic (|FC| ≥ 2, *p* < 0.05) analyses. (**B**) The expression distribution of the transcriptome in 12 samples (*n* = 4) from three porcine muscle tissues. (**C**) PCA composition diagrams of transcriptomes and proteomes in three types of porcine muscle tissues. (**D**) Sample correlation analysis of 12 porcine samples. (**E**–**G**) Volcano plots of differentially expressed genes from pairwise comparisons among three tissue types. (**H**) The number of differentially expressed genes or proteins from pairwise comparisons among the three porcine muscle tissues. (**I**–**K**) Expression analysis, PCA, and sample correlation analysis in the three mouse tissue types (*n* = 3). (**L**–**N**) Volcano plots of differentially expressed genes from pairwise comparisons among the three tissue types. (**O**) The number of differentially expressed genes or proteins from pairwise comparisons among the three muscle tissues in mice.

Muscle tissue is classified by its structure and function into smooth, cardiac, and skeletal muscle. However, comprehensive studies on the evolutionary conservation of molecular differences among these three muscle tissues have been limited. In porcine and murine models, we addressed this gap using multi-omics sequencing (Figure 1A). Principal component analysis (PCA) of the transcriptome and proteome in pigs, along with inter-sample correlation, revealed greater variation among the three tissue groups than within each group. Notably, compared with the intestine, which is mainly composed of smooth muscle, cardiac and skeletal muscles were positioned closer to each other in the PCA plot (Figure 1B–D), suggesting higher molecular similarity between them. Pairwise comparisons of the three tissues based on transcriptomic and proteomic data demonstrated substantial differences at both transcriptional and translational levels (Figure 1E–H; see Tables S1 and S2 for a complete gene list), with cardiac and skeletal muscles exhibiting relatively similar expression profiles. Consistent trends were observed in mice (Figure 1I–O; see Tables S3 and S4 for a complete gene list). These findings lay the groundwork for the subsequent identification of tissue-specific gene expression patterns.

### 2.2. Transcriptomic and Proteomic Analysis to Identify Enriched Genes in Mammalian Striated Muscle

**Figure 2:**
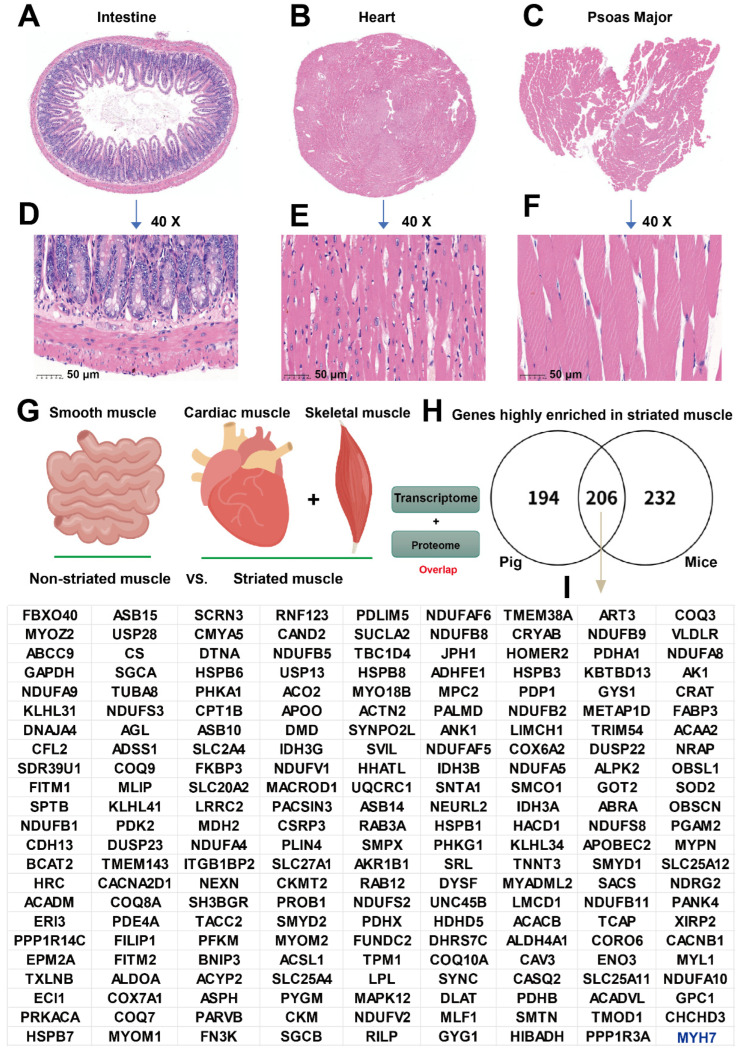
Multi-omics screening of striated muscle-enriched genes in pigs and mice. (**A**–**C**) H&E staining of the intestine, heart, and psoas muscle from 8-week-old male mice. (**D**–**F**) Higher-magnification (40×) views of the three tissues. Scale bar = 50 μm. (**G**) Workflow for identifying striated muscle-enriched genes by integrating porcine and murine transcriptomic (|FC| ≥ 1.2, *p* < 0.05) and proteomic (|FC| ≥ 2, *p* < 0.05) data. (**H**,**I**) Venn diagram depicting the 206 conserved striated muscle-enriched genes identified in both species, including *MYH7*.

As noted in previous sections, muscle tissues are divided into striated and non-striated types. Striated muscle includes cardiac muscle and skeletal muscle, whereas non-striated muscle refers to smooth muscle, which is distributed throughout internal organs such as the intestine. H&E staining of the three muscle tissue types confirmed accurate sampling: the intestine exhibited villi, whereas the heart and psoas muscle displayed distinct striations, verifying the reliability of our samples for subsequent analysis (Figure 2A–F). As shown in Figure 2G, the experimental design was as follows: to identify genes enriched in striated muscle, we compared striated versus non-striated muscle tissues from pigs and mice at both the transcriptome (|FC| ≥ 1.2 and *p* < 0.05) and proteome (|FC| ≥ 2 and *p* < 0.05) levels and determined the overlap between these datasets. Through comprehensive analysis, we identified 206 conserved (Figure 2H), enriched genes in striated muscle across both species (Figure 2I). *MYH7* was excluded due to high variation in psoas muscle samples (*p* ≥ 0.05 vs. intestine), although its expression was still higher in psoas than in the intestine, supporting its status as a striated muscle-enriched gene (see Table S8 for a complete gene list). The detection of known striated muscle markers (e.g., *FBXO40*, *MYOZ2*, and *DMD*) within this gene set provides strong internal validation for the accuracy of our sequencing data.

### 2.3. Identification of Enriched Genes in Cardiac, Skeletal, and Smooth Muscles

**Figure 3:**
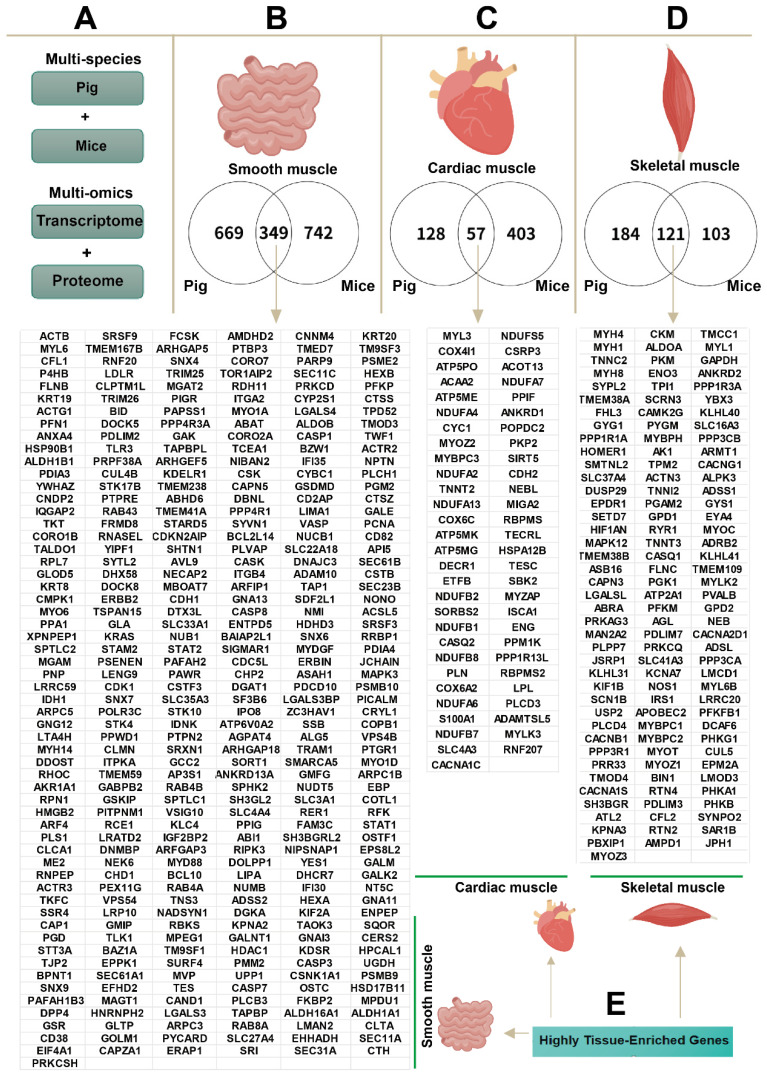
Identification of enriched genes in cardiac, skeletal, and smooth muscles. (**A**) The schematic diagram depicts an experimental design that employs both mouse and pig models, combined with transcriptomic (|FC| ≥ 1.2, *p* < 0.05) and proteomic (|FC| ≥ 2, *p* < 0.05) data, to identify tissue-enriched genes. (**B**) Venn diagram of intestinal-enriched genes conserved across species. (**C**) Venn diagram of cardiac-enriched genes conserved across species. (**D**) Venn diagram of skeletal muscle-enriched genes conserved across species. (**E**) Allocation diagram of enriched genes across intestinal, cardiac, and skeletal muscle.

We employed an integrated cross-species (pig and mouse) and multi-omics (transcriptomics and proteomics) analytical strategy to screen for tissue-specific, enriched genes in smooth muscle (intestine), cardiac muscle, and skeletal muscle (Figure 3A). The results showed that, compared to the two striated muscle types (skeletal and cardiac), 349 enriched genes were identified in the intestine (Figure 3B). Compared to the other two tissues, 57 enriched genes were identified in cardiac muscle (Figure 3C), and 121 enriched genes were identified in skeletal muscle (Figure 3D). These tissue-specific, enriched genes were systematically categorized (Figure 3E; see Table S8 for a complete gene list). Notably, the intestine contained the highest number of specific genes (349), followed by skeletal muscle (121), with cardiac muscle having the fewest (57).

### 2.4. KEGG and GO Analysis of Enriched Genes in Skeletal, Cardiac, and Smooth Muscles

**Figure 4:**
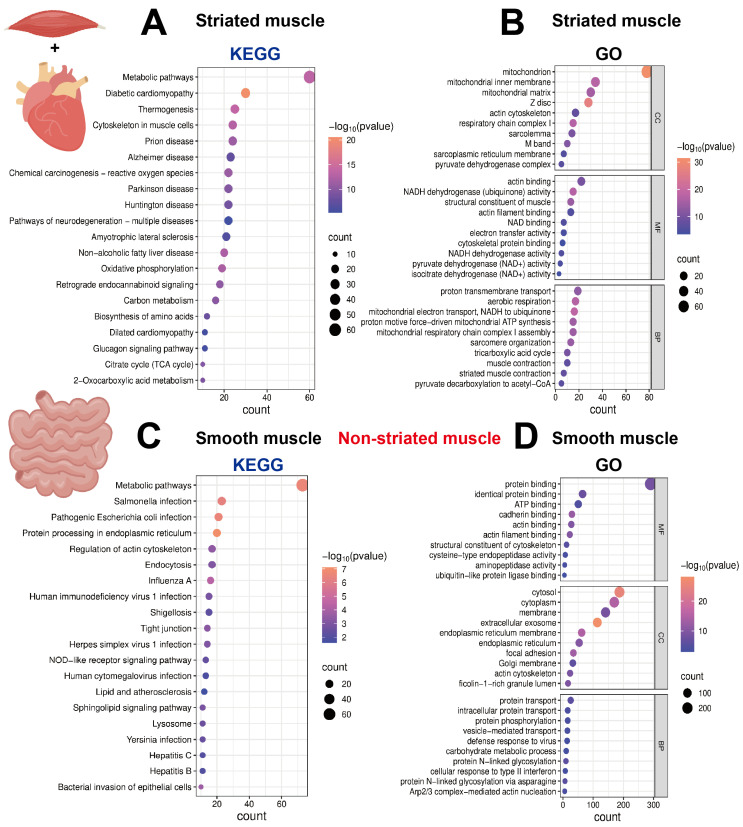
KEGG and GO enrichment analysis of enriched genes in striated and smooth muscles. (**A**,**B**) KEGG and GO enrichment analysis of 207 genes that are conserved in both mice and pigs and enriched in striated muscle. (**C**,**D**) KEGG and GO enrichment analysis of 349 genes that are conserved in both mice and pigs and enriched in smooth muscle.

To investigate the signaling pathways enriched for the corresponding genes in the three muscle tissue types, we performed enrichment analyses using the Kyoto Encyclopedia of Genes and Genomes (KEGG) and GO databases. The results of KEGG enrichment analysis of both striated and smooth muscles showed that the top enriched pathway was “Metabolic pathways”, reflecting the fundamental processes essential for all cellular life. However, their functional pathways diverged significantly: striated muscle was specifically enriched in the “Cytoskeleton in muscle cells” pathway, directly corresponding to its myofiber contraction function; in comparison, smooth muscle was enriched in the “Regulation of actin cytoskeleton” pathway, primarily involved in cell morphology maintenance and intercellular connections. In summary, both KEGG and GO enrichment results indicate that the core function of striated muscle centers on energy metabolism and contraction, whereas intestinal smooth muscle focuses on barrier defense and immunity (Figure 4A–D).

Further comparison within striated muscle using KEGG analysis revealed that cardiac muscle and skeletal muscle shared commonalities in pathways related to basic contractile structures, calcium signaling, and neurodegenerative diseases. However, their core differences were pronounced: cardiac muscle pathways were highly concentrated in oxidative phosphorylation and thermogenesis, highlighting its absolute dependence on efficient aerobic metabolism as a continuous pumping organ. In contrast, skeletal muscle pathways were widely enriched in glucose metabolism (e.g., glycolysis and gluconeogenesis), amino acid biosynthesis, and various hormone signaling pathways, reflecting its central role in systemic energy distribution, substrate metabolism, and hormonal response. Additionally, cardiac muscle was specifically associated with metabolic complication pathways such as diabetic cardiomyopathy, whereas skeletal muscle prominently featured the “Motor proteins” pathway related to active movement.

**Figure 5:**
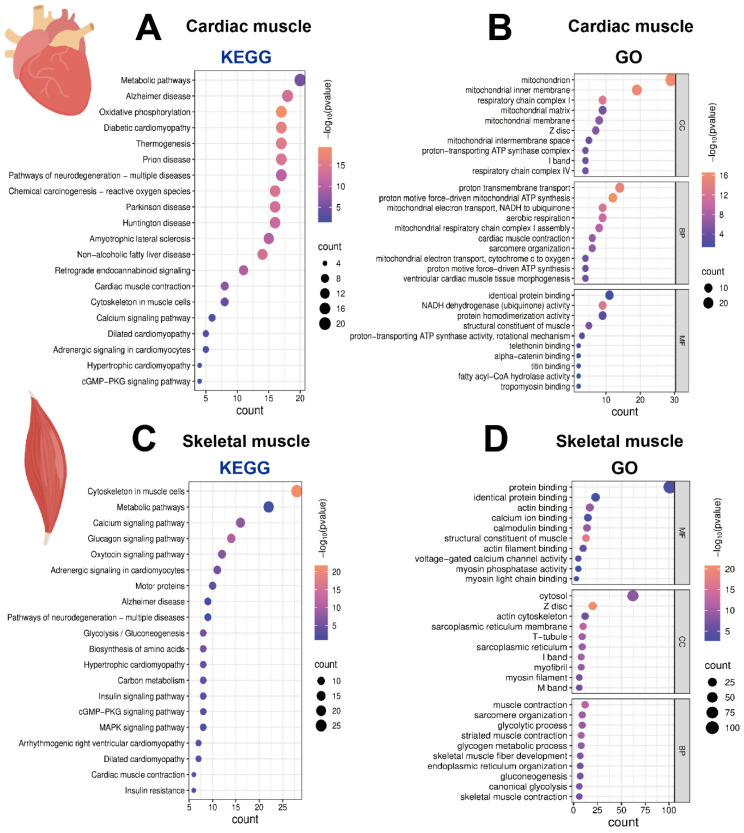
KEGG and GO enrichment analysis of enriched genes in cardiac and skeletal muscles. (**A**,**B**) KEGG and GO enrichment analysis of 57 genes that are conserved in both mice and pigs and enriched in cardiac muscle. (**C**,**D**) KEGG and GO enrichment analysis of 121 genes that are conserved in both mice and pigs and enriched in skeletal muscle.

At the GO enrichment level, the analysis further elucidated the functional differentiation between cardiac muscle and skeletal muscle from the perspectives of subcellular structures, molecular functions, and biological processes. Genes in cardiac muscle were markedly and almost exclusively enriched in terms related to mitochondrial compartments, oxidative phosphorylation, and proton transport, perfectly aligning with its physiological demand for “unlimited endurance” as a continuously working organ. In contrast, genes in skeletal muscle, while maintaining basic sarcomere structures, were broadly enriched in terms such as glycogen metabolism, glycolysis, voltage-gated calcium channel activity, and myosin activity regulation, demonstrating the metabolic flexibility and precise neural control evolved to accommodate voluntary, rapid, and variable-intensity movements. The two muscle types only shared commonalities in the basic contractile structures and actin-binding functions of striated muscle (Figure 5A–D).

### 2.5. Identification of Enriched Metabolites in Cardiac, Skeletal, and Smooth Muscles

**Figure 6:**
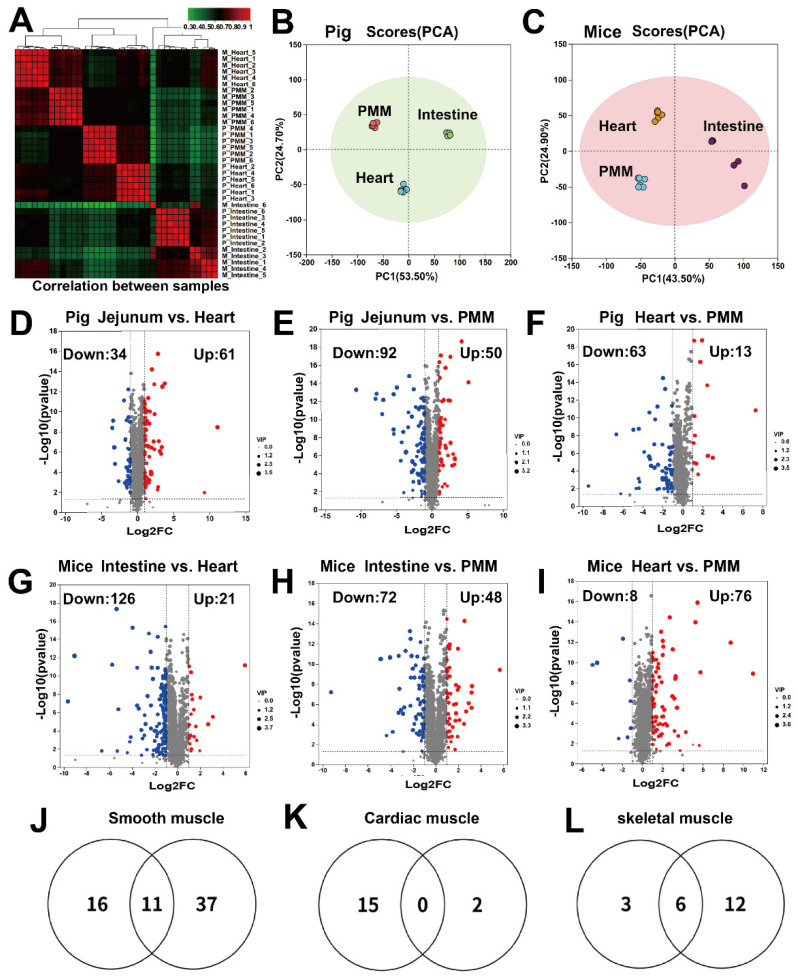
Identification of highly enriched metabolites in porcine and murine cardiac, skeletal, and smooth muscles. (**A**) Sample correlation analysis of 36 samples from the three tissue types in pigs and mice. (**B**,**C**) Principal component analysis (PCA) of the three muscle tissues from pigs and mice. (**D**–**F**) Volcano plots of pairwise comparisons among the three muscle tissues in pigs. (**G**–**I**) Volcano plots of pairwise comparisons among the three muscle tissues in mice. (**J**–**L**) Screening of metabolites that are conserved between pigs and mice and highly enriched in smooth, cardiac, and skeletal muscles.

**Figure 7:**
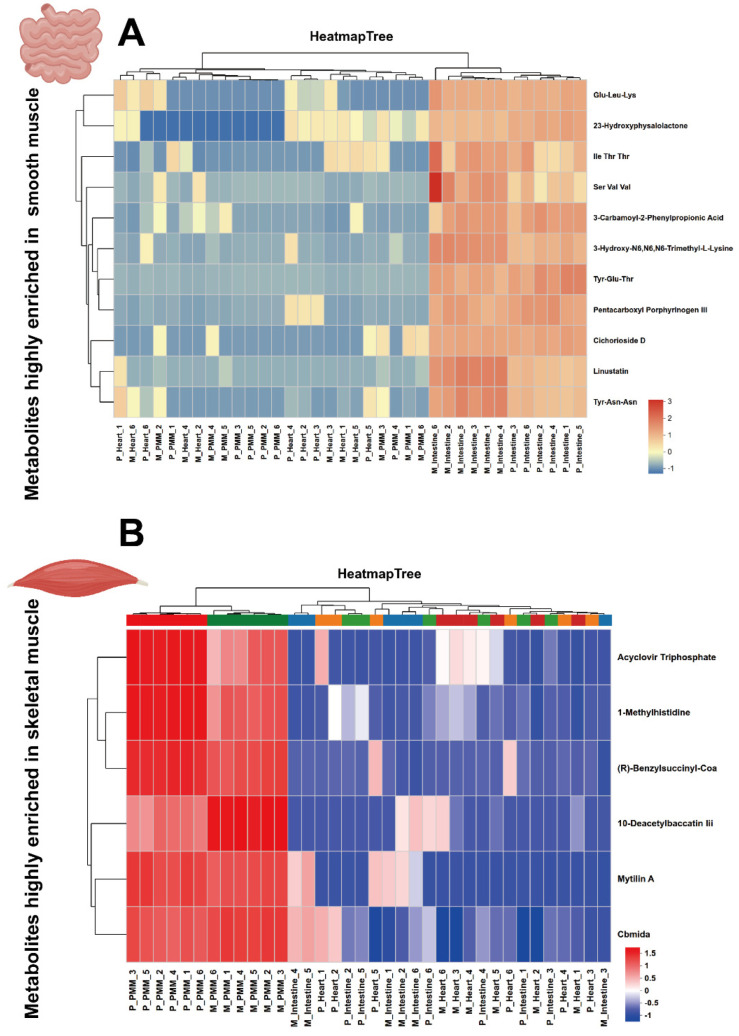
Profiling of enriched metabolites in intestine and skeletal muscle types across pigs and mice. (**A**) List of 11 metabolites highly enriched in the intestine. (**B**) List of 6 metabolites highly enriched in skeletal muscle.

Following comparative analysis of the transcriptome and proteome across the three muscle tissue types, we further conducted LC-MS-based metabolomics to systematically investigate their metabolic differences. To ensure data reliability, all metabolomic samples from pigs and mice were processed in a single batch to minimize instrumental variation. The results of inter-sample correlation and principal component analysis (PCA) revealed greater between-group than within-group variation, with cardiac muscle and skeletal muscle clustering more closely in the metabolomic space, indicating higher metabolic similarity between these two tissues compared to intestinal muscle (Figure 6A–C). Using a stringent fold-change threshold (|FC| ≥ 2, *p* < 0.05), we identified multiple differentially abundant metabolites in pairwise comparisons among the three tissues in both species (Figure 6D–I; see Tables S5 and S6 for a complete metabolite list). By intersecting results across species, we ultimately identified 11 metabolites conserved and enriched in the intestine (Figure 6J), 0 metabolites conserved and enriched in the cardiac muscle (Figure 6K) and 6 metabolites conserved and enriched in the skeletal muscle (Figure 6L). The tissue-enrichment pattern at the metabolomic level was largely consistent with that observed in the transcriptome: the intestine contained the highest number of tissue-specific metabolites, followed by skeletal muscle, with cardiac muscle having the fewest. These results further validate the reliability and consistency of our data. Analysis of Figure 6 led to the identification of 11 metabolites highly enriched in the intestine (Figure 7A) and 6 metabolites enriched in skeletal muscle (Figure 7B).

### 2.6. Comparative Analysis of Metabolites Conserved Across Species in Fast-Twitch vs. Slow-Twitch Muscle

**Figure 8:**
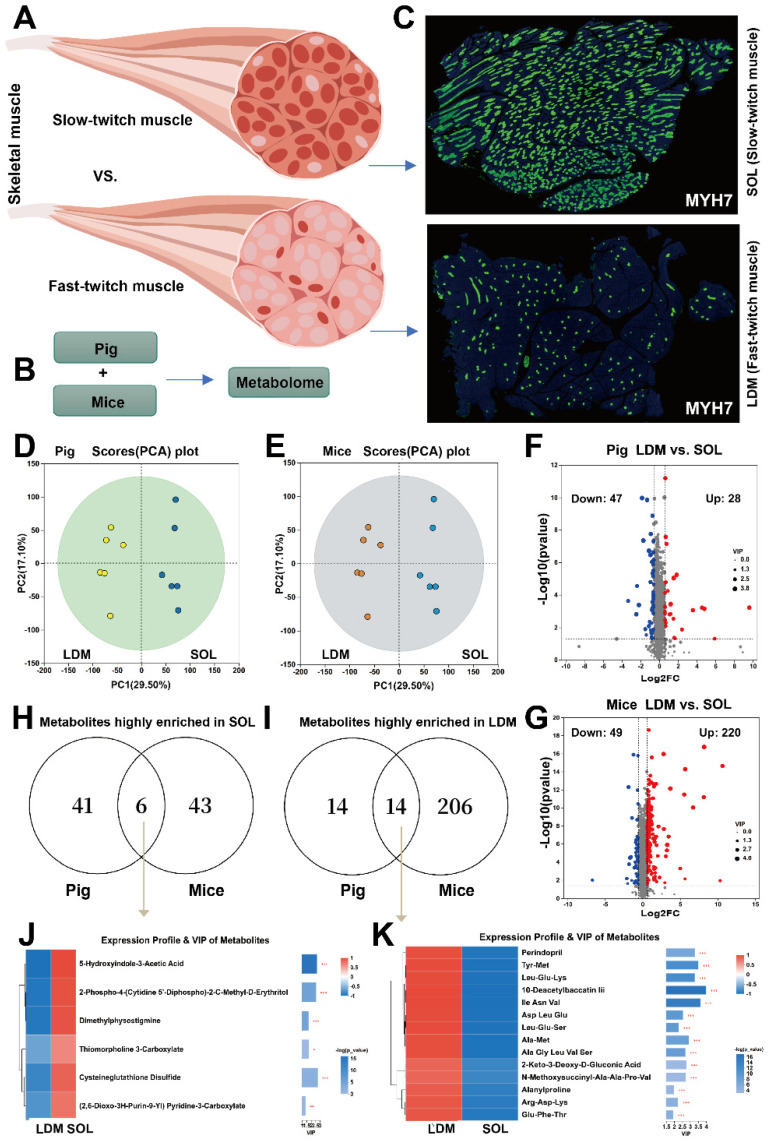
Screening for conserved differential metabolites in fast-twitch and slow-twitch muscles across pigs and mice. (**A**) Two extreme types of skeletal muscle fibers: fast-twitch and slow-twitch fibers. (**B**) Schematic diagram of the experimental setup: metabolomic analysis of fast and slow muscles from adult pigs and mice. (**C**) Immunofluorescence staining of porcine *soleus* muscle and *longissimus dorsi* muscle (LDM) using an antibody against the slow muscle marker gene MYH7, showing the proportion of slow muscle fibers relative to the total muscle fibers. (**D**,**E**) Principal component analysis plot of the metabolomic profiles of the two muscle fiber types in pigs and mice, *n* = 6. (**F**,**G**) Differential metabolites in porcine and murine fast-twitch fibers vs. slow-twitch fibers (|FC| ≥ 2, VIP > 1, and *p* < 0.05). (**H**,**I**) Screening of conserved genes in fast-twitch and slow-twitch muscles. (**J**) Six metabolites associated with slow-twitch fibers that are conserved in both pigs and mice. (**K**) Fourteen metabolites associated with fast-twitch fibers that are conserved in both pigs and mice. * *p* < 0.05; ** *p* < 0.01; *** *p* < 0.001.

Skeletal muscle constitutes approximately 40% of adult mammalian body mass. A systematic understanding of its metabolism is therefore crucial for comprehending muscle tissues. While the authors of previous studies have explored metabolic differences among smooth, cardiac, and skeletal muscles, they did not address the heterogeneity within skeletal muscle, which comprises slow-twitch and fast-twitch fiber types (Figure 8A). To resolve these distinct metabolic profiles, we focused on the *longissimus dorsi* (LDM, fast-twitch muscle) and *soleus* (SOL, slow-twitch muscle) muscles (Figure 8B). MYH7 antibody staining revealed that SOL was slow-twitch, whereas the LDM was fast-twitch, confirming accurate sampling (Figure 8C). PCA results showed low intra-group variation but distinct inter-group differences between pigs and mice, validating the high quality of the data (Figure 8D–G; see Table S7 for a complete metabolite list). The 6 slow-twitch muscle-related metabolites are listed in Figure 8J, and the 14 fast-twitch muscle-related metabolites are presented in Figure 8K. Our cross-species metabolic map provides a resource for understanding muscle physiology and potential targets for improving meat quality.

## 3. Discussion

In living organisms, cells are the fundamental units. These cells, which share common origins and functions, together with the extracellular matrix, constitute tissues [15]. Muscle tissue, as a major tissue type, is classified by its structure and function into smooth, cardiac, and skeletal muscle. We hypothesize that muscle tissues exhibit greater molecular similarity to each other than to non-muscle tissues (e.g., adipose tissue). Although in-depth research on individual muscle tissue within a single species, such as across 27 developmental stages of porcine skeletal muscle [16], has been conducted, studies on the evolutionary conservation of molecular differences across all three muscle tissue types remain limited. Therefore, we employed pigs and mice as models to profile the transcriptome, proteome, and metabolome of these three muscle tissues, thereby defining their molecular landscapes. Furthermore, we specifically investigated skeletal muscle heterogeneity through metabolomic analysis of fast-twitch and slow-twitch fibers.

Supporting our hypothesis, PCA results revealed clear group separation, affirming data quality, and showed the close proximity between cardiac muscle and skeletal muscle, aligning with their classification as striated muscles [17]. These clear molecular distinctions provide a foundation for screening genes specific to smooth, cardiac and skeletal muscles. Strikingly, we unexpectedly identified 207 genes with high expression at both the mRNA and protein levels in striated muscles (compared to smooth muscle), most of which were undetectable in smooth muscle or other organs. The specific and coordinated expression of these genes at both transcriptional and translational levels provides compelling evidence that they play fundamental roles in the biology of striated muscles. Among these genes, Dystrophin (*DMD*), the largest human gene [18], is a master regulator of striated muscle. Its protein product is critical for structural and functional integrity, and consequently, its mutations lead to severe disorders, including Duchenne and Becker muscular dystrophy [19]. Furthermore, we identified other genes critical for striated muscle structure and integrity, such as *MYH7* [20], *CSRP3* [21], and *MYOM2* [22], which validates the accuracy of our systematic screening. In light of this coordinated expression, we hypothesize that other genes in the set are functionally important in striated muscle. The findings of a subsequent literature review confirmed that the functions of several genes in this context, particularly in skeletal muscle, remain poorly characterized. Notably, *LRRC2*, which is implicated in cardiac mitochondrial function and hypertrophy [23], has no reported function in skeletal muscle. Similarly, *PPP1R14C* has been identified as a candidate gene for muscle development [24]; however, it still lacks functional characterization. Based on these findings, we will focus future work on investigating the roles of *LRRC2* and *PPP1R14C* in skeletal muscle. This striated muscle-specific gene set thus presents valuable new targets for cardiac and skeletal muscle research.

The functional differences between skeletal muscle and cardiac muscle are driven by their distinct molecular profiles: high *MYH7* expression in the heart versus a complex mix of myosin isoforms in skeletal muscle, suggesting greater heterogeneity in the latter. Our analysis confirms this pattern: genes such as *MYH1*, *MYH4*, and *ACTN3* are specific to skeletal muscle; in contrast, *MYOM2*, *MYL3*, and *TECRL* are cardiac-enriched and show minimal expression in skeletal muscle. Our hypothesis is reinforced by the disparity in DEG numbers: 121 genes were highly enriched in skeletal muscle compared to only 57 in cardiac muscle. While some of these genes are expressed in both tissues, their expression levels diverge, reflecting functional specialization. We also identified several understudied genes in striated muscle, most notably *LRRC20*, which is enriched in skeletal muscle but less abundant in cardiac muscle [25]. This finding prompted us to initiate functional validation studies, which are now nearing completion. It should be noted that a limitation of this study is the fact that we did not incorporate variables such as developmental stage and physiological condition [26,27], which are known to influence gene expression. In a separate comparative analysis, we found 349 genes enriched in the smooth muscle (intestine) compared to striated muscle. Some of these genes were gut-specific, whereas others were shared with the liver, indicating potential molecular crosstalk between these digestive organs [28]. Collectively, our study provides a framework for understanding the molecular distinctions between muscle tissues.

The results of our cross-species metabolomic analysis revealed 11 conserved highly enriched metabolites in the intestine, 6 in skeletal muscle, and none in the heart, supporting the greater molecular complexity of skeletal versus cardiac muscle. Most functions of these metabolites are unknown. Notably, 10-Deacetylbaccatin III was enriched in skeletal muscle and was also highly enriched in fast-twitch fibers, suggesting that it is a high-profile target for future functional studies. This finding may have translational relevance for animal science, potentially informing strategies in breeding and meat quality improvement. Our findings also address the diversity of muscle tissues based on distinct myosin heavy chain isoforms. Smooth muscle specifically expresses *MYH11* [29]; in comparison, cardiac muscle mainly expresses *MYH7* and *MYH6*. Skeletal muscle, crucial for its body-wide distribution and substantial mass, accounting for approximately 40% of body weight in mammals [7], comprises two major fiber subtypes: slow-twitch and fast-twitch [30]. The fast-to-slow fiber ratio in skeletal muscle is influenced by external factors such as diet [31]. Consequently, fiber composition is markedly heterogeneous across anatomical regions and developmental stages [32]. Understanding this heterogeneity has important implications for regenerative medicine, combating age-related muscle decline, and improving livestock product quality. Although conventional and single-cell sequencing have substantially advanced our understanding of fiber type differences [33], the authors of most studies have focused on transcriptional and epigenetic regulation. By contrast, comparative metabolomic studies across species remain limited, prompting our interest. In the present study, we selected the slow-twitch-dominated soleus and fast-twitch-dominated longissimus dorsi muscle from pigs and mice for LC-MS analysis. Applying a high fold-change threshold, we identified 6 slow-twitch-enriched and 14 fast-twitch-enriched metabolites. Among them, most have unknown functions; however, one notable finding is the fact that 5-Hydroxyindole-3-acetic acid (5-HIAA), the main metabolite of serotonin, is enriched in soleus. As serotonin mediates vasoconstriction and platelet activation [34], this enrichment suggests a greater metabolic capacity in the soleus than in the longissimus dorsi. Interestingly, metabolites enriched in fast-twitch fibers were predominantly small peptides, which may be related to their high rate of protein synthesis and degradation. Regarding the specific functions of the other metabolites, further research is required.

The core strength of this work lies in integrating proteomics with transcriptomics and metabolomics, a combination scarce in existing studies. By analyzing all three layers from the same samples, we bridge gene expression to metabolic phenotype through the direct measurement of protein translation. We employed a cross-species comparison between pig and mouse models, despite their known differences in muscle composition and basal metabolism. This approach was designed to eliminate species-specific traits and highlight evolutionarily conserved pathways that may underpin fundamental aspects of muscle tissues. We focused on the qualitative overlap in significantly enriched pathways and genes, rather than direct quantitative comparisons. Two main limitations should be noted in this study. First, we focused our analysis on only two mammalian models and thus could not reveal the evolutionary characteristics of birds or fish in muscle tissues. Furthermore, while conventional sequencing technologies were employed, functional studies using gene editing were not conducted to validate the candidate genes. In future studies, multiple advanced omics techniques (e.g., single-cell RNA sequencing and ATAC sequencing) should be integrated to resolve cellular heterogeneity. In summary, the results of this study provide new gene and metabolite targets for muscle biology and agricultural animal breeding.

## 4. Materials and Methods

### 4.1. Experimental Animals

All animal experiments conducted in this study were approved by the Ministry of Agriculture of China and the Committee of Animal Care at Yunnan Agricultural University (permit number: CACAYU20240519; permit date: 19 May 2024). In this study, we used six male half-sibling pigs and six male half-sibling C57BL/6 mice. The pigs were housed in a standardized farm environment with free access to water and fed a diet meeting NRC nutritional standards. Ambient conditions were maintained at a temperature of 25 ± 2 °C and relative humidity of 60–70%, with 6 h of natural light provided daily. Sampling was performed when the pigs reached 180 days of age. The mice were kept in a standard animal facility and euthanized for sampling at 8 weeks of age. From all animals, tissue samples, including the heart, *psoas major* muscle, *soleus* muscle, *longissimus dorsi* muscle, and intestine, were collected. All samples were immediately snap-frozen in liquid nitrogen and stored at −80 °C for subsequent transcriptomic, proteomic, and metabolomic analyses.

### 4.2. Bulk RNA-Sequencing and Data Analysis

In this study, transcriptomic profiles of 21 samples from the three muscle tissues in two species were generated by means of bulk RNA-seq. RNA extraction and quality control were performed using TRIzol^®^ Reagent (Invitrogen, Carlsbad, CA, USA). RNA integrity and concentration were assessed with an Agilent 5300 Bioanalyzer (Agilent, CA, USA) and NanoDrop ND-2000 (Thermo Fisher Scientific, Waltham, MA, USA), respectively. Samples were retained only if they met the following thresholds: concentration ≥ 20 ng/μL, total RNA > 1 μg, and RQN > 4.5. Libraries were prepared by Shanghai Majorbio Bio-pharm Biotechnology Co., Ltd. (Shanghai, China). Poly (A) mRNA was enriched using oligo (dT) beads, fragmented, and reverse-transcribed into cDNA with random hexamers. After second-strand synthesis, cDNA underwent end repair, A-tailing, and adapter ligation (Illumina^®^ Stranded mRNA Prep) (Illumina, CA, USA). Libraries were size-selected (300–400 bp), PCR-amplified, and sequenced on an Illumina platform. Raw reads were trimmed and filtered with fastp before alignment to the reference genome using HISAT2 (http://ccb.jhu.edu/software/hisat2/index.shtml, accessed on 2 December 2025) in orientation-aware mode. Transcripts were assembled and quantified with StringTie (v2.2.3). Gene expression was quantified by means of RSEM (TPM-normalized). Differential expression analysis was performed with DESeq2/DEGseq, with DEGs defined as |fold change| ≥ 1.2 and *p*adj < 0.05. Functional enrichment of DEGs was analyzed using the GO and KEGG databases. Inter-sample correlation was assessed using Pearson correlation. Biological replicates showed higher correlation than across groups. Hierarchical clustering was subsequently performed using complete linkage with Euclidean distance. Orthologous genes were defined based on one-to-one orthology lists from Ensembl (pig vs. mouse) to support functional conservation comparisons.

### 4.3. DIA Proteomic Sequencing and Data Analysis

Proteomic analysis was performed on the same 21 samples by Shanghai Majorbio Bio-Pharm Technology Co., Ltd. (Shanghai, China). Proteins were extracted from frozen tissues using BPP buffer, followed by phenol extraction and ammonium acetate–methanol precipitation. Protein concentration was measured via BCA assay. For digestion, 100 µg of protein was reduced with TCEP (Invitrogen, Carlsbad, CA, USA), alkylated with IAM (Sigma, St. Louis, MO, USA), and digested overnight with trypsin. Peptides were desalted using HLB cartridges and quantified by means of NanoDrop UV absorbance. DIA data were processed in Spectronaut using a metagenomic protein database. Parameters included trypsin digestion, carbamidomethylation as a fixed modification, oxidation and acetylation as variable modifications, and FDR ≤ 0.01. Quantification was performed with MaxLFQ. Differential expression analysis involved an unpaired *t*-test in R. Proteins with a fold change > 2 and a *p*-value < 0.05 were defined as DEPs.

### 4.4. LC-MS Analysis

A total of 60 samples from five tissues (heart, *psoas major*, *soleus*, *longissimus dorsi*, and intestine) across pigs and mice (*n* = 6 per group) were subjected to LC-MS analysis by Shanghai Majorbio Bio-Pharm Technology Co., Ltd. (Shanghai, China). Metabolites were extracted from 50 mg tissue with 400 µL methanol–water (4:1, *v*/*v*) containing 0.02 mg/mL L-2-chlorophenylalanine as an internal standard. After homogenization and sonication, proteins were precipitated at −20 °C. The supernatant was collected following centrifugation. A pooled QC sample was analyzed intermittently throughout the run. LC-MS was performed on an AB SCIEX UPLC-TripleTOF system. Raw data were processed using Progenesis QI, with peaks from the internal standard and artifacts excluded. Metabolites were identified by querying the HMDB, Metlin, and Majorbio databases. Data were filtered to retain features present in ≥80% of samples per group, with missing values filled by the feature minimum, followed by total-sum normalization. Features with QC RSD > 30% were removed, and data were log10-transformed prior to statistical analysis. PCA and OPLS-DA were conducted using the ropls R package (v1.6.2), with model validity assessed through 7-fold cross-validation. Metabolites with VIP > 1 (OPLS-DA) and *p* < 0.05 (Student’s *t*-test) were considered significantly altered.

### 4.5. Immunofluorescence Staining Experiment

Immunofluorescence was performed on fresh soleus and longissimus dorsi muscles. The tissues were fixed in 4% paraformaldehyde, paraffin-embedded, and sectioned at 20 μm thickness with a cryostat. The sections were then incubated overnight at 4 °C with an anti-slow-twitch myosin heavy chain primary antibody (GB112131, Servicebio, Wuhan, China) and subsequently incubated for 1 h at room temperature with fluorescent secondary antibodies. Imaging was conducted using a fluorescence microscope.

### 4.6. H&E Staining Experiment

We performed H&E staining on deparaffinized sections based on the instructions of a commercial kit, which included staining, dehydration, and mounting. The prepared sections were observed and imaged under a microscope. For morphometric analysis, the cross-sectional area of muscle fibers was quantified from the acquired images using ImageJ 2 software (National Institutes of Health, Bethesda, MD, USA), and a comparative analysis was conducted on the measurements.

## 5. Conclusions

In this multi-species, multi-omics study, we defined the molecular expression profiles of muscle tissues. We identified 207 genes that are enriched in striated muscle. Distinct sets of genes and metabolites were specifically enriched in each tissue: skeletal muscle (121 genes and 6 metabolites), cardiac muscle (57 genes and no specific metabolites), and smooth muscle (349 genes and 11 metabolites). Within skeletal muscle, 14 fast-twitch and 6 slow-twitch fiber-enriched metabolites were identified. These results provide a valuable molecular foundation for muscle tissue research.

## Data Availability

The original contributions presented in this study are included in the article/Supplementary Material. Further inquiries can be directed to the corresponding author.

## Supplementary Materials

The following supporting information can be downloaded at: https://www.mdpi.com/article/10.3390/ijms27010242/s1.

## References

[1] Brunello E., Fusi L. (2024). Regulating Striated Muscle Contraction: Through Thick and Thin. Annu. Rev. Physiol..

[2] Cheng L.K., O’GRady G., Du P., Egbuji J.U., Windsor J.A., Pullan A.J. (2010). Gastrointestinal system. Wiley Interdiscip. Rev. Syst. Biol..

[3] Larsson L., Degens H., Li M., Salviati L., Lee Y.I., Thompson W., Kirkland J.L., Sandri M. (2019). Sarcopenia: Aging-Related Loss of Muscle Mass and Function. Physiol. Rev..

[4] Sweeney H.L., Hammers D.W. (2018). Muscle Contraction. Cold Spring Harbor Perspect. Biol..

[5] McCarthy N., Tie G., Madha S., He R., Kraiczy J., Maglieri A., Shivdasani R.A. (2023). Smooth muscle contributes to the development and function of a layered intestinal stem cell niche. Dev. Cell.

[6] Steinhauser M.L., Lee R.T. (2011). Regeneration of the heart. Embo Mol. Med..

[7] Frontera W.R., Ochala J. (2014). Skeletal muscle: A brief review of structure and function. Calcif. Tissue Int..

[8] Maggi L., Mavroidis M., Psarras S., Capetanaki Y., Lattanzi G. (2021). Skeletal and Cardiac Muscle Disorders Caused by Mutations in Genes Encoding Intermediate Filament Proteins. Int. J. Mol. Sci..

[9] Hou C., Jiang X., Zhang H., Zheng J., Qiu Q., Zhang Y., Sun X., Xu M., Chang A.C.Y., Xie L. (2022). TECRL deficiency results in aberrant mitochondrial function in cardiomyocytes. Commun. Biol..

[10] Geng S., Liu S.-B., He W., Pan X., Sun Y., Xue T., Han S., Lou J., Chang Y., Zheng J. (2024). Deletion of TECRL promotes skeletal muscle repair by up-regulating EGR2. Proc. Natl. Acad. Sci. USA.

[11] Shi J., Luo L., Eash J., Ibebunjo C., Glass D.J. (2011). The SCF-Fbxo40 complex induces IRS1 ubiquitination in skeletal muscle, limiting IGF1 signaling. Dev. Cell.

[12] Zou Y., Li Z., Zou Y., Hao H., Li N., Li Q. (2018). An FBXO40 knockout generated by CRISPR/Cas9 causes muscle hypertrophy in pigs without detectable pathological effects. Biochem. Biophys. Res. Commun..

[13] LOngaro L., Zhou X., Wang Y., Schultz H., Zhou Z., Buddle E.R.S., Brûlé E., Lin Y.-F., Schang G., Hagg A. (2025). Muscle-derived myostatin is a major endocrine driver of follicle-stimulating hormone synthesis. Science.

[14] Tan B., Hong L., Xiao L., Wu J., Lu G., Wang S., Liu L., Zheng E., Cai G., Li Z. (2025). Rewiring of 3D chromatin topology orchestrates transcriptional reprogramming in muscle fiber-type specification and transformation. Nat. Commun..

[15] AchTheocharis A.D., Skandalis S.S., Gialeli C., Karamanos N.K. (2016). Extracellular matrix structure. Adv. Drug Deliv. Rev..

[16] Yang Y., Yan J., Fan X., Chen J., Wang Z., Liu X., Yi G., Liu Y., Niu Y., Zhang L. (2021). The genome variation and developmental transcriptome maps reveal genetic differentiation of skeletal muscle in pigs. PLoS Genet..

[17] Wittenberg J.B., Wittenberg B.A. (2003). Myoglobin function reassessed. J. Exp. Biol..

[18] Le S., Yu M., Hovan L., Zhao Z., Ervasti J., Yan J. (2018). Dystrophin as a Molecular Shock Absorber. Acs Nano.

[19] Liu S., Su T., Xia X., Zhou Z.H. (2024). Native DGC structure rationalizes muscular dystrophy-causing mutations. Nature.

[20] de Frutos F., Ochoa J.P., Webster G., Jansen M., Remior P., Rasmussen T.B., Sabater-Molina M., Barriales-Villa R., Girolami F., Cesar S. (2024). Clinical Features and Outcomes of Pediatric MYH7-Related Dilated Cardiomyopathy. J. Am. Heart Assoc..

[21] Cui C., Han S., Tang S., He H., Shen X., Zhao J., Chen Y., Wei Y., Wang Y., Zhu Q. (2020). The Autophagy Regulatory Molecule CSRP3 Interacts with LC3 and Protects Against Muscular Dystrophy. Int. J. Mol. Sci..

[22] Auxerre-Plantié E., Nielsen T., Grunert M., Olejniczak O., Perrot A., Özcelik C., Harries D., Matinmehr F., Dos Remedios C., Mühlfeld C. (2020). Identification of MYOM2 as a candidate gene in hypertrophic cardiomyopathy and Tetralogy of Fallot, and its functional evaluation in the Drosophila heart. Dis. Model. Mech..

[23] McDermott-Roe C., Leleu M., Rowe G.C., Palygin O., Bukowy J.D., Kuo J., Rech M., Hermans-Beijnsberger S., Schaefer S., Adami E. (2017). Transcriptome-wide co-expression analysis identifies LRRC2 as a novel mediator of mitochondrial and cardiac function. PLoS ONE.

[24] Miao W., Ma Z., Tang Z., Yu L., Liu S., Huang T., Wang P., Wu T., Song Z., Zhang H. (2021). Integrative ATAC-seq and RNA-seq Analysis of the Longissimus Muscle of Luchuan and Duroc Pigs. Front. Nutr..

[25] Ehrlich K.C., Lacey M., Ehrlich M. (2020). Epigenetics of Skeletal Muscle-Associated Genes in the ASB, LRRC, TMEM, and OSBPL Gene Families. Epigenomes.

[26] Liufu S., Lan Q., Liu X., Chen B., Xu X., Ai N., Li X., Yu Z., Ma H. (2023). Transcriptome Analysis Reveals the Age-Related Developmental Dynamics Pattern of the Longissimus Dorsi Muscle in Ningxiang Pigs. Genes.

[27] Talman V., Teppo J., Pöhö P., Movahedi P., Vaikkinen A., Karhu S.T., Trošt K., Suvitaival T., Heikkonen J., Pahikkala T. (2018). Molecular Atlas of Postnatal Mouse Heart Development. J. Am. Heart Assoc..

[28] Gao Y., Jin Q., Gao C., Chen Y., Sun Z., Guo G., Peng J. (2022). Unraveling Differential Transcriptomes and Cell Types in Zebrafish Larvae Intestine and Liver. Cells.

[29] Fournier N., Fabre A. (2022). Smooth muscle motility disorder phenotypes: A systematic review of cases associated with seven pathogenic genes (ACTG2, MYH11, FLNA, MYLK, RAD21, MYL9 and LMOD1). Intractable Rare Dis. Res..

[30] Gong H.M., Ma W., Regnier M., Irving T.C. (2022). Thick filament activation is different in fast- and slow-twitch skeletal muscle. J. Physiol..

[31] Chen X., Guo Y., Jia G., Liu G., Zhao H., Huang Z. (2018). Arginine promotes skeletal muscle fiber type transformation from fast-twitch to slow-twitch via Sirt1/AMPK pathway. J. Nutr. Biochem..

[32] Wang K., Li X., Liu X., Liufu S., Xiao L., Chen B., Chen W., Jiang J., Liu Y., Ma H. (2025). Multi-Omics Insights into Postnatal Skeletal Muscle Development in Duroc Pigs. Animals.

[33] LuMa L., Meng Y., An Y., Han P., Zhang C., Yue Y., Wen C., Shi X., Jin J., Yang G. (2024). Single-cell RNA-seq reveals novel interaction between muscle satellite cells and fibro-adipogenic progenitors mediated with FGF7 signalling. J. Cachexia Sarcopenia Muscle.

[34] Jeong Y.-M., Li H., Kim S.Y., Park W.-J., Yun H.-Y., Baek K.J., Kwon N.S., Jeong J.H., Myung S.C., Kim D.-S. (2011). Photo-activated 5-hydroxyindole-3-acetic acid induces apoptosis of prostate and bladder cancer cells. J. Photochem. Photobiol. B.

